# Into the light: the rise of shadowbox simulation to illuminate decision-making

**DOI:** 10.1186/s41077-026-00450-x

**Published:** 2026-06-04

**Authors:** Victoria Ruth Tallentire, Lachlan Dick, Robert Hart, Thalia Monro-Somerville, Benjamin Clarke, Russell Allan

**Affiliations:** 1 Medical Education Directorate, NHS Lothian, Edinburgh, Scotland; 2 Medical Directorate, NHS Education for Scotland, Edinburgh, Scotland; 3 Scottish Centre for Simulation and Human Factors, NHS Forth Valley, Larbert, Scotland; 4 Anaesthetics and Critical Care Directorate, NHS Greater Glasgow and Clyde, Glasgow, Scotland; 5 Critical Care Directorate, NHS Lothian, Edinburgh, Scotland; 6 Emergency Medicine and Critical Care, NHS Lothian, Edinburgh, Scotland; 7 Medical Education Directorate, NHS Greater Glasgow and Clyde, Glasgow, Scotland

**Keywords:** ShadowBox method, Shadowbox simulation, Cognitive skills, Cognitive transformation theory, Naturalistic decision-making, Sports psychology

## Abstract

**Background:**

Shadowbox simulation is an educational approach that utilises the ShadowBox method, originally developed in high-stakes fields such as firefighting and the military, adapted for healthcare education. Grounded in cognitive transformation theory and drawing heavily on naturalistic decision-making, shadowbox simulation makes expert reasoning visible and accessible, enabling learners to compare their decision-making with that of experts and develop complex cognitive and behavioural skills.

**Main body:**

Early applications of shadowbox simulation in healthcare have demonstrated feasibility across diverse contexts. Building on these foundations, we describe the implementation of a hybrid shadowbox simulation format within Scotland’s national Internal Medicine Training program. This model combines video vignettes of expert practice, structured decision-points, group discussion and procedural practice, all supported by expert facilitation. Evaluation across more than 40 sessions shows that learners value the safe learning environment, the opportunity to embrace uncertainty and explore multiple perspectives, and the balance of reflective discussion with procedural refreshers. Feedback indicates that the format may foster deeper engagement, greater knowledge retention, and broader participation compared with traditional immersive simulation, while requiring fewer resources. By synthesising evidence from outside healthcare, reviewing recent adaptations, and presenting the principles and outcomes of national implementation, this article positions shadowbox simulation as a theory-informed, resource-efficient, and scalable method that advances simulation practice.

**Conclusion:**

We argue that shadowbox simulation offers particular promise for developing non-technical or behavioural skills, supporting reflective learning, and widening access to simulation-based education. Future potential applications extending to leadership, delegation, and induction training across healthcare professions are discussed.

**Graphical Abstract:**

**Figure:**
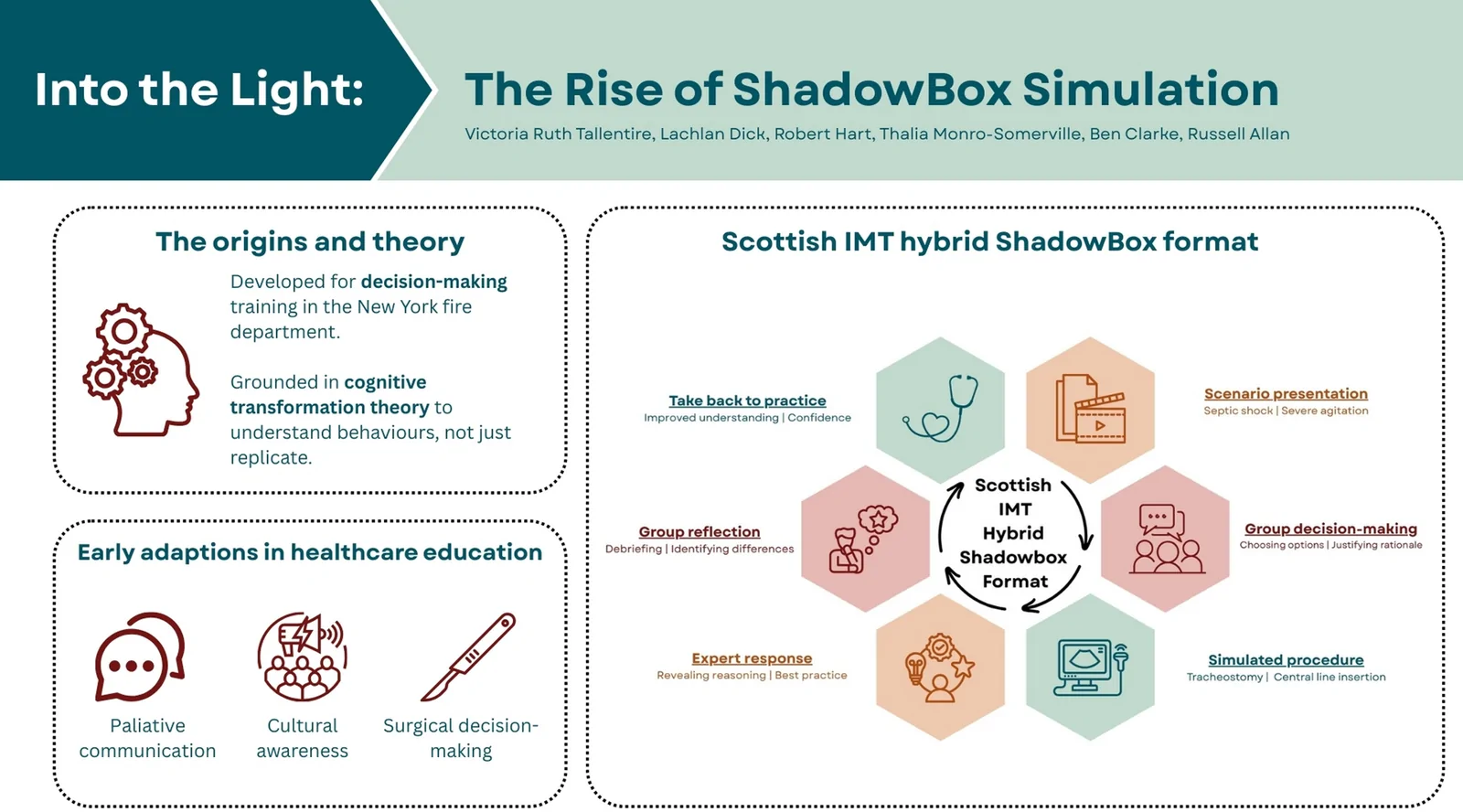

## Background

Simulation-based education has expanded beyond manikin-based scenarios to encompass diverse approaches for developing technical and behavioural skills. As the field matures, there is increasing recognition that advancing simulation practice requires not only innovative techniques, but also theoretically informed, evidence-grounded strategies that can be applied across contexts [1]. Inspired by the practice of shadowboxing in sport and originating in high-stakes decision-making domains such as firefighting and the military, the ShadowBox method represents one such approach. By making expert reasoning visible and accessible to learners, the educational concept of shadowboxing supports the development of complex cognitive and behavioural skills that are otherwise difficult to teach. This article synthesises the theoretical foundations, early applications, and emerging adaptations of shadowboxing in healthcare simulation, and illustrates its use within a national medical simulation program through a novel hybrid format. In doing so, we aim to highlight the principles underpinning its success, its practical utility for simulation professionals, and its potential to shape future simulation practice.

### Origin of the cognitive ShadowBox method

Although the cognitive training approach ‘ShadowBox’ was developed in decision-making research rather than sport, its name draws on the long-established boxing practice of shadowboxing. In boxing, shadowboxing refers to training against an imagined rather than physically present opponent, allowing the athlete to rehearse punches, footwork, defence, distance, and timing in a purposeful but simulated encounter [2]. Coaching guidance emphasises that effective shadowboxing depends on keeping an opponent ‘in the mind’s eye’, sometimes using a mirror or drawn outline to simulate the absent adversary.

Building on these conceptual foundations, the cognitive training method originally known as ‘ShadowBox’ was conceived in 2008 from the Masters’ thesis of Neil Hintze, a retired Fire Battalion Chief in New York. It was subsequently co-developed with cognitive psychologist Gary Klein and was used to train New York fire department personnel, enabling learners to compare their decision-making with expert reasoning in realistic scenarios [3].

**Fig. 1:**
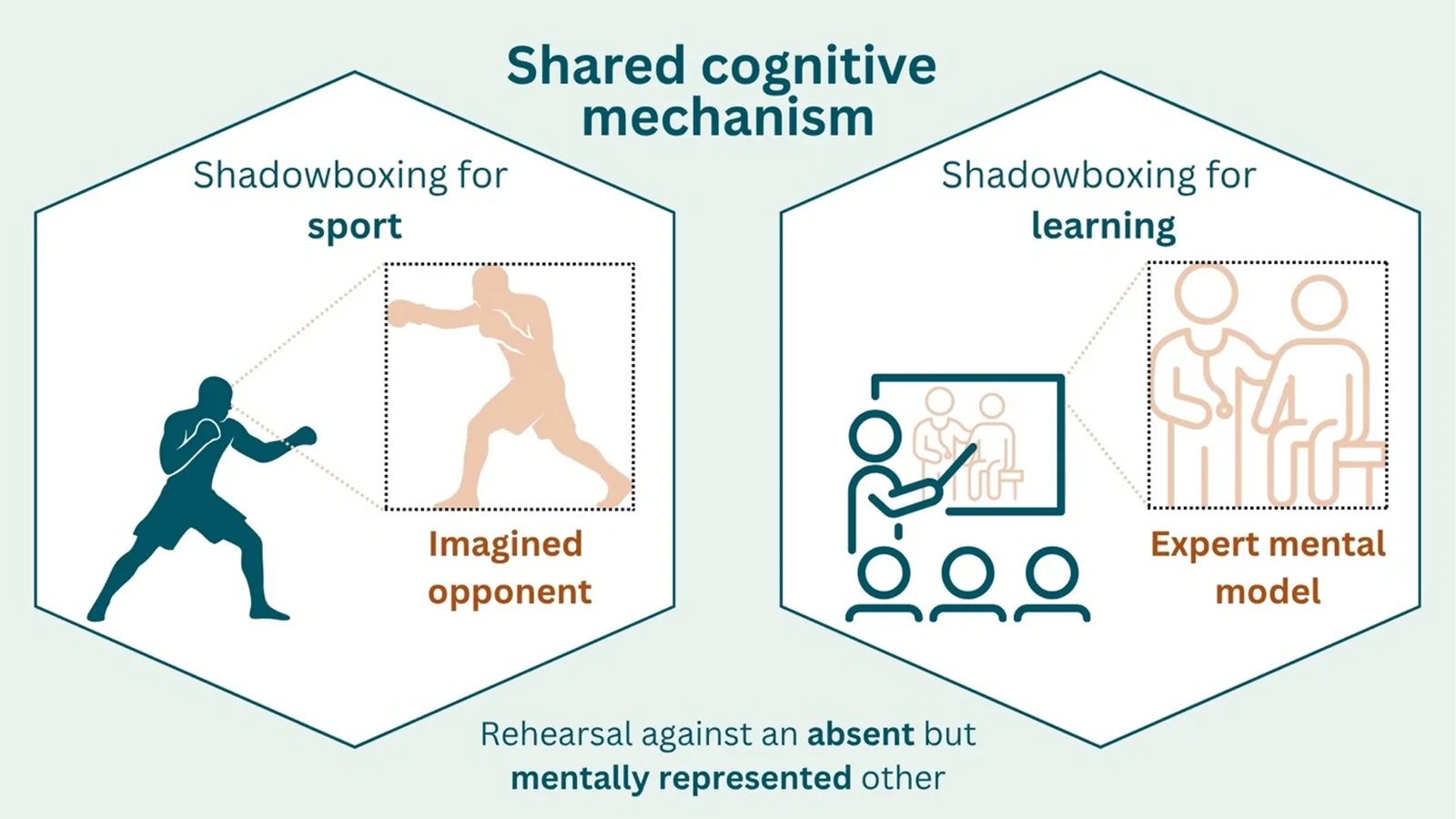
The shared cognitive mechanism underpinning shadowboxing in sport and learning

The ShadowBox training method is scenario-based cognitive instruction that teaches tacit decision-making using experts’ mental models. The three key features of the method are: scenario presentation with decision-points where learners choose options and justify their rationale; revealing of experts’ reasoning for the same points; and opportunity for learners to reflect on differences between their decisions and experts’. The approach allows learners to “see through the expert’s eyes” without experts needing to be physically present. Parallelling the concept of shadowboxing in sport (Fig. 1), the ShadowBox training method allows learners to refine performance through low-stakes rehearsal in relation to an imagined but realistic counterpart, making tacit judgement and response more visible and more open to deliberate refinement.

### Theoretical basis

Shadowboxing, the educational concept underlying the original ShadowBox training method, is best understood as a method for making the otherwise tacit processes of expert cognition explicit, thereby allowing novice to compare their reasoning with experts and develop institution that is grounded in experience and understood in context. Making expert cognition explicit is important because it is often an opaque process for learners; they may observe what is done without understanding how the expert has interpreted cues, prioritised competing concerns, recognised uncertainty, or decided when to reconsider an initial judgement. By rendering those underlying cognitive moves visible, shadowboxing can support learners to understand the reasoning processes that make adaptive and context-sensitive performance possible in challenging conditions.

In Klein and colleagues’ account, the method is influenced by cognitive transformation theory (CTT), which emphasises changes in mental models and frames, including the recognition and revision of flawed assumptions, rather than the simple accumulation of facts or procedures [4, 5]. Shadowboxing operationalises this by asking learners to engage with realistic scenarios, articulate their reasoning, and then compare it with the prioritisation, cue use, and rationale of experts. In this sense, the method is designed to expose and refine the learner’s underlying way of understanding a situation, not merely their overt behavioural response.

The method draws heavily on the naturalistic decision-making framework (NDM), which studies how experts make rapid, high-stakes decisions in contexts characterised by uncertainty [6]. Within NDM, recognition-primed decision models suggest that experts often do not begin by comparing multiple options in an abstract, exhaustive manner. Instead, they recognise familiar patterns, generate a plausible course of action, and mentally simulate it to test feasibility and likely consequences. Importantly, however, this should be read as a descriptive account of one form of expert performance in context, not as a normative claim that good decision-making always relies on rapid intuition or that analytic comparison is unnecessary [7].

This distinction matters in health professions education. Dual-process accounts of clinical reasoning propose that expert judgement may involve both fast, pattern-based processing and slower, more deliberate analytic review [8, 9]. Educationally, the goal is therefore not simply to reproduce an expert’s initial pattern match, but to help learners recognise what experts notice, how they frame the problem, and when they check, revise, or override an initial impression. That point is particularly salient in safety-critical settings, where fixation and premature closure can contribute to harm; for example, airway literature on unrecognised oesophageal intubation explicitly highlights the need to reconsider an initial interpretation and avoid fixation error [10]. A strength of shadowboxing, therefore, is not that it teaches uncritical intuition, but that it can be used to surface both intuitive and reflective aspects of expert judgement, including the cues, priorities, and moments of reconsideration that support safer decision-making. For this reason, shadowboxing may be particularly useful for developing complex non-technical or behavioural skills, such as communication, prioritisation, situational awareness, and decision-making, where effective performance depends not only on observable action but also on how practitioners interpret situations and revise their understanding in context [11].

### Early uses of shadowboxing

One of the earliest applications of shadowboxing was in firefighting in 2008 (Hintze’s Master’s project). Newly promoted officers were presented with a series of challenging scenario-based exercises coupled with materials illustrating the ‘expert mindset’ (collated from 14 experienced fire department officers) allowing them to see the scenarios through the eyes of experienced officers.

To assess their subsequent decision-making performance, the newly promoted officers were presented with written or video-based firefighting scenarios at key decision points. For each scenario, they had to choose a course of action from several plausible options and provide a rationale for their choice. Their responses were then compared with expert panels’ rankings and justifications, with scoring based on how closely the decisions and reasoning aligned with those of experienced officers. Improved performance was demonstrated by greater convergence with expert reasoning after training compared with the matched control group [3].

Subsequently shadowboxing methodology was used to train additional groups of fire-fighters, along with police and military officers as part of the ‘Strategic Social Interaction Modules’ (SSIM). The aim of the SSIM program was to explore why, in the context of warfare, some police and military personnel were particularly skilled at securing voluntary compliance from civilians. The findings were used to develop interpersonal skills training for those groups, with studies demonstrating significant improvements in learners’ abilities to match expert reasoning during tactical decision-making exercises [3].

### Experience within health professions education

While the ShadowBox method was originally conceived as a cognitive training tool in high-stakes domains, its adaptation into shadowbox simulation within health professions education has introduced important differences. The classic ShadowBox method presents learners with decision points and systematically compares their choices and rationales to those of experts, with performance judged by alignment. In contrast, shadowbox simulation applies these principles within the broader pedagogy of simulation-based education. Video vignettes or simulated encounters are typically combined with facilitated discussion or debriefing, and the emphasis is often on reflective exploration rather than scoring against expert responses. Thus, while both approaches aim to make tacit expert cognition visible, shadowbox simulation blends these insights with established simulation practices to create a more flexible and context-sensitive learning experience.

In 2020, Harder & Turner described a novel shadowbox simulation, whereby novice nursing students were exposed to expert communication in a palliative context [12]. They therefore demonstrated the feasibility of the approach for developing non-technical or behavioural skills without high resource demands. Within Scotland, experience of shadowbox simulation dates to 2021, when a week-long elective course on infectious diseases due to be hosted in Edinburgh was moved online due to COVID-19 travel restrictions. Modifications to the approach described by Harder & Turner were required to facilitate the use of shadowboxing for synchronous online distance learning. Consequently, Mutch & Oliver developed a video-based simulation to demonstrate an expert approach to taking an effective travel history from an unwell patient [13]. Following completion of the scenario, the expert was available online to discuss their thought-processes and provide feedback to learners.

Following further adaptation, MacLennan et al. used shadowboxing principles in 2024 to design video resources that could be used for synchronous debriefing, personal reflective study or small group learning on topics relating to palliative care [14]. Leveraging routinely generated video, shadowbox simulation has also been proposed as a potential tool for decision-making training in operating theatre environments [15] and utilised to raise cultural awareness in nursing studies [16]. In recent work, footage taken from television broadcasts has been adapted for shadowbox simulation in primary care, greatly reducing the resource required to develop content [17].

### Implementation in a national simulation program

The Scottish Internal Medicine Training (IMT) simulation program has been running since 2019 and spans the three years of basic training as a physician in Scotland, prior to specialization [18]. The two-day ‘registrar ready’ course in the third year of the program is critical to ensuring that the resident doctors feel able to take the next significant step in their training pathway. Feedback from early iterations of the course suggested that round table sessions discussing theoretical clinical decisions were not well-received by learners, and further immersive simulation was requested. However, concurrent use of the immersive simulation equipment and facilities for parallel sessions within the same program meant that these requests could not be met.

**Fig. 2:**
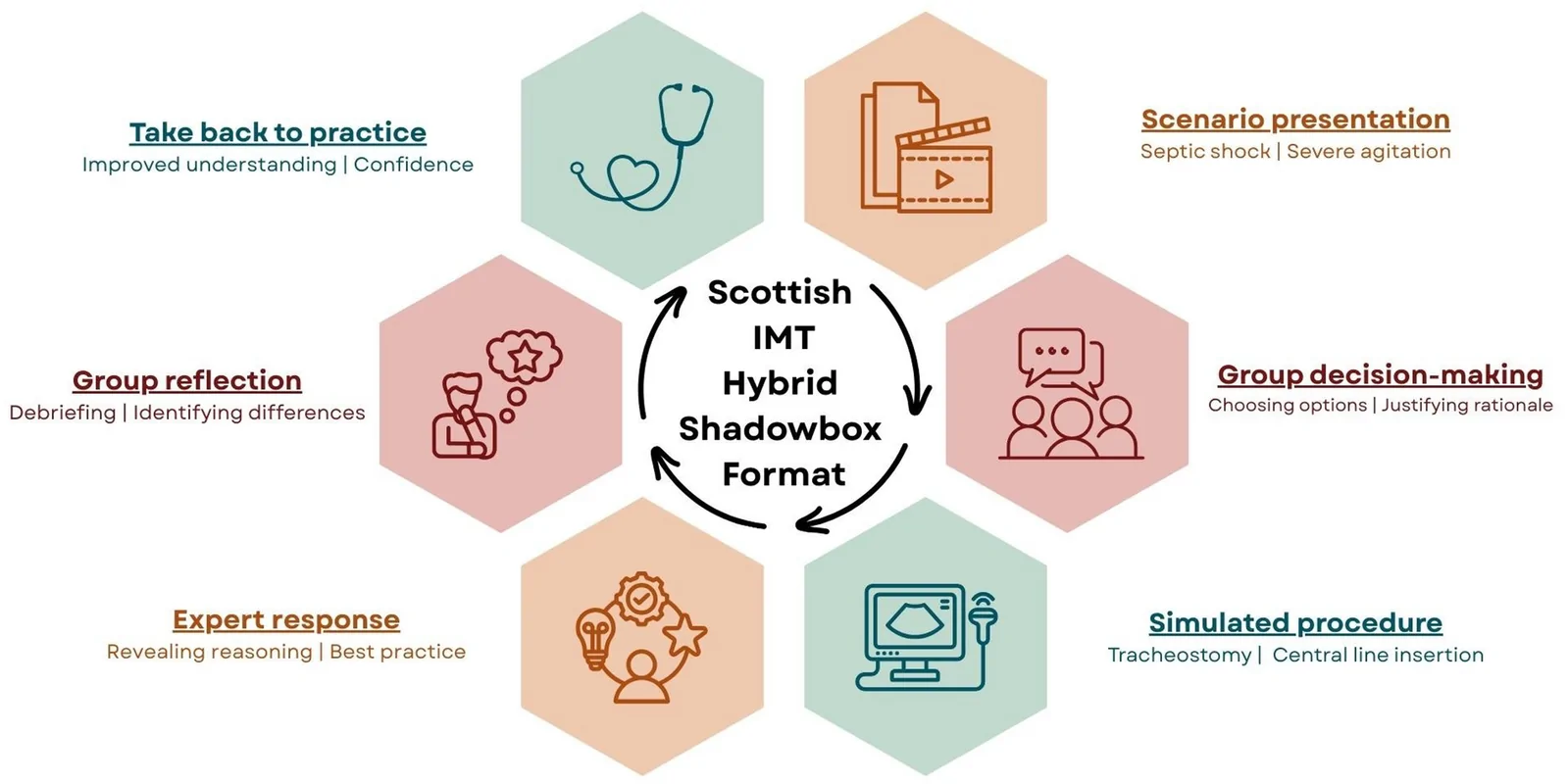
The Scottish IMT hybrid shadowbox simulation format

Consequently, a modified shadowboxing approach was utilised to develop and pilot sessions incorporating video footage of experienced doctors managing a range of clinical encounters (e.g. septic shock, severe agitation). Clinical scenarios were divided into short video clips of several minutes, with each segment focussed on a particular intended learning outcome to guide a stepwise approach to the encounter. During pauses between video clips, questions were posed to small groups of learners, in a similar format to a debriefing following an immersive simulation scenario [19]. When a procedure was required within a case (e.g. central venous catheterisation or management of tracheostomy emergencies), learners undertook the procedure on a part task trainer, before returning to the debriefing conversation and video. This innovative approach therefore became known as the ‘hybrid shadowbox simulation’ format (Fig. 2), integrating video exemplars and the lens of an expert from the shadowboxing with expert facilitation and group reflection from immersive simulation.

### Evaluation of hybrid shadowbox simulation

Following the first seven hybrid shadowbox simulation sessions involving 40 learners, feedback showed that many learners preferred it to the more familiar immersive simulation approach that they had encountered earlier in the program. They valued the opportunity to acknowledge uncertainty and discuss a variety of options. The peer reflection fostered a sense of shared experience and purpose, whilst procedural refreshers allowed engagement to be maintained throughout the two-hour sessions. The nature of the sessions also particularly appealed to learners who found immersive simulation highly anxiety-provoking and facilitation (given the content of the videos was familiar prior to the session) was less cognitively burdensome for faculty. This feedback from learners, along with the enthusiasm evident at a national simulation conference (winner of the best oral presentation at the Association for Simulated Practice in Healthcare conference in November 2024) [20], encouraged us to embed the sessions in a more permanent way within the longitudinal simulation program.

Between February and June 2025, 36 hybrid shadowbox simulation sessions were delivered to groups of 4–6 learners as part of the ‘registrar ready’ course. Free text comments from 95 learners reiterated the findings from the pilot phase that the format provided a safe and less anxiety-provoking learning environment compared with traditional immersive simulation, allowing them to engage in open discussion without the pressure of performance.

Learners valued the opportunity to explore ambiguity, share different perspectives, and contrast their reasoning with expert input, which was seen as enhancing information retention and reinforcing key physiological principles. The hybrid design (combining video vignettes, facilitated discussion, and practical tasks) was considered engaging and effective for consolidating both cognitive and procedural skills. Exposure to ‘gold standard’ management, opportunities to discuss both effective and suboptimal approaches, and the chance to learn from colleagues across different contexts were frequently described as strengths. Although a minority questioned whether video added value beyond seminar-style teaching, the majority regarded shadowbox simulation as a refreshing and effective complement to immersive simulation.

From a faculty perspective, the shadowbox simulation sessions offered several important benefits over the more traditional immersive, full-scenario approaches that are used elsewhere in the program. The ability to openly critique, analyse and, at times, disagree with the management of an expert contrasted with conversations in immersive simulation debriefings, when contesting the decisions of a peer could be socially awkward or divisive. This likely reflected both the modality and the learning culture it enabled. By shifting the focus from critique of a peer’s live performance to discussion of an externalised expert response, the format may have reduced interpersonal risk and social threat. However, such openness also depends on facilitation that promotes psychological safety and supports an educational alliance in which learners feel able to voice uncertainty and dissent [21, 22]. Clinical cases were presented in short, digestible video clips, allowing a period of reflection after each one. This encouraged focused discussion on specific aspects of the case, as opposed to the post-scenario debriefings in immersive simulation, which tend to cover broader topics or focus disproportionally on particular timepoints within the scenario. Throughout the sessions, faculty observed that the peer discussions drew on the experiences of the doctors training across different specialty areas, demonstrating the shared decision making expected in clinical practice.

Questions of fidelity and authenticity are also relevant to this approach. In hybrid shadowbox simulation, the educational aim is not to recreate every physical and sensory aspect of clinical practice, but to provide sufficient authenticity for learners to engage credibly with the underlying judgement, prioritisation, and procedural steps required in context. Therefore, functional authenticity may be more important than high physical fidelity [23]. For the procedural components, the level of realism required depends on the intended learning outcome; where the goal is orientation to sequence, decision-making, and discussion of management, lower-fidelity procedural rehearsal may be sufficient, but when the goal is refinement of psychomotor technique, more realistic simulation may be needed. This suggests that authenticity does matter, but its value lies in how well the simulation aligns with the specific cognitive, behavioural, and procedural objectives of the session. In our context, the procedural refreshers appeared to function primarily as a means of sustaining engagement and reinforcing key management steps, rather than as a substitute for high-fidelity skills training.

### Comparisons to similar methods

Shadowbox simulation shares features with several other decision-making training approaches but differs in some important ways. Tactical Decision Games (TDGs), first used in the military but also applied in healthcare, involve teams being given rapidly evolving scenarios to test recognition and response under pressure [24]. Like shadowboxing, TDGs foster situational awareness, but typically emphasise speed and improvisation rather than structured comparison with expert thought processes [25]. Case-based discussions, widely used in undergraduate and postgraduate health professions education (e.g. in problem-based learning curricula), also present learners with scenarios and decision points, but these are usually instructor-led and exploratory, whereas shadowboxing systematically integrates expert rankings and rationales to make tacit knowledge visible [13].

Mental rehearsal and decision-making exercises, such as Visually Enhanced Mental Simulation (VEMS), encourage learners to think through complex problems and anticipate outcomes, but do not provide the same opportunity for benchmarking decisions against expert cognition [26]. Finally, cognitive apprenticeship models in healthcare education, such as bedside teaching in internal medicine, align closely with the shadowboxing approach of using expert demonstration and reflection, though usually require direct expert–novice interaction and are limited by problems of accessibility and scale. Although shadowbox simulation requires significant effort to produce and record the video clips, thereafter it requires only a single facilitator per session with no additional resource development.

Beyond these simulation-adjacent approaches, shadowbox simulation can also be situated within wider learning theory. It shares some features with case-based clinical reasoning which uses authentic cases to connect knowledge with practice and often relies on facilitators to model expert thinking [27]. However, in many case-based discussions, expert reasoning remains implicit, contingent on the individual facilitator, and variably articulated. In contrast, shadowbox simulation deliberately externalises expert judgement through structured rankings, rationales and explicit comparison with learner responses, thereby making tacit cognition more consistently visible. Shadowbox simulation also has some resonance with script-based approaches such as script concordance testing, in that both draw on expert panels and reasoning in conditions of uncertainty [28]. However, script concordance is principally an assessment method, whereas shadowbox simulation uses expert reasoning formatively to stimulate discussion, reflection, and refinement of judgement. Thus, while shadowbox simulation draws on principles common to TDGs, case-based learning, script concordance testing, mental rehearsal and cognitive apprenticeship, it is distinctive in making expert mental models explicit and transferable in a structured, resource-efficient, scalable way.

### Future applications

Shadowbox simulation, in the hybrid format that is used within IMT in Scotland, has been an efficient and effective way to meet the needs of our learners. The extent of its possible application within healthcare education is exciting. The key ingredients can be included in various quantities for different contexts and learners. For example, our hybrid model incorporated face-to-face expert debriefing, but the method has been utilised with an expert present remotely [13], or without the presence of an expert at all [14]. Regardless of the specific format, learners can engage with expert mental models through videos and written materials, which often reveal subtle decision-making cues and reasoning that are rarely verbalised. The potential for shadowbox simulation also reaches beyond direct clinical scenarios. Within the IMT simulation program, we are considering its broader application in relation to topics such as leadership, delegation and team communication. For other groups such as medical students, video-based approaches for induction to clinical environments have the strengths of convenience and the option of re-watching. By incorporating decision-points, discussion and reflection, additional learning could be achieved with little extra upfront resources needed.

## Conclusions

This article has outlined the theoretical foundations, empirical origins and contemporary adaptations of shadowbox simulation, illustrating how a method first developed to capture expert decision-making has been translated into healthcare education. Shadowbox simulation makes tacit expertise visible and accessible to learners, supporting the development of complex non-technical or behavioural skills. Through piloting and implementation in the context of a national simulation program, we demonstrate a hybrid format that has been well received by learners seeking safe, engaging, and reflective learning opportunities. By synthesising evidence from other fields, reviewing early healthcare applications, and presenting practical principles for adaptation, we argue that shadowbox simulation represents a scalable, resource-efficient, theory-informed approach that advances current simulation practice and has broad potential across simulation contexts.

## Data Availability

The datasets used and/or analysed during the current study are available from the corresponding author on reasonable request.

## Abbreviations

CTT: Cognitive Transformation Theory
NDM: Naturalistic Decision-making Framework
SSIM: Strategic Social Interaction Modules
COVID-19: Coronavirus Disease 2019
IMT: Internal Medicine Training
TDG: Tactical Decision Game
VEMS: Visually Enhanced Mental Simulation
